# Differential Regulation of L-Arginine Metabolism through NOS2 and Arginases during Infection with *Trypanosoma cruzi*

**DOI:** 10.3390/pathogens13100878

**Published:** 2024-10-08

**Authors:** Arturo A. Wilkins-Rodríguez, Paz María Salazar-Schettino, Rebeca G. Manning-Cela, Laila Gutiérrez-Kobeh

**Affiliations:** 1 Unidad de Investigación UNAM-INC, División de Investigación, Facultad de Medicina, Universidad Nacional Autónoma de México-Instituto Nacional de Cardiología “Ignacio Chávez”, Mexico City 14080, Mexico; wilkins_aar@comunidad.unam.mx; 2 Laboratorio de Biología de Parásitos, Departamento de Microbiología y Parasitología, Facultad de Medicina, Universidad Nacional Autónoma de México, Mexico City 04510, Mexico; pazmar@unam.mx; 3 Departamento de Biomedicina Molecular, Centro de Investigación y de Estudios Avanzados del IPN, Mexico City 07360, Mexico; rmanning@cinvestav.mx

**Keywords:** arginases 1 and 2, cardiomyocytes, L-arginine, macrophages, NOS2, *Trypanosoma cruzi*

## Abstract

L-arginine metabolism through arginases and inducible nitric oxide synthase (NOS2) constitutes a fundamental axis for the resolution or progression of Chagas disease. Infection with *Trypanosoma cruzi* can cause a wide spectrum of disease, ranging from acute forms contained by the host immune response to chronic ones, such as the chronic chagasic cardiomyopathy. Here, we analyzed, in an in vitro model, the ability of two *T. cruzi* isolates, with different degrees of virulence, to regulate the metabolism of L-arginine through arginase 1 (Arg-1) and NOS2 in macrophages and through arginase 2 (Arg-2) and NOS2 in cardiomyocytes. Stimulation of bone marrow-derived macrophages (BMMΦ), obtained from CD1 mice, with TNF-α + IFN-γ induced their polarization into classically activated macrophages (CAMΦ), which expressed functional NOS2, while stimulation with IL-4 induced their polarization into alternatively activated macrophages (AAMΦ), which expressed functional Arg-1. Interestingly, stimulation of cardiomyocytes, obtained from hearts of CD1 neonatal mice, with TNF-α + IFN-γ or IL-4 also resulted in functional NOS2 and arginase expression, as observed in CAMΦ and AAMΦ, but Arg-2 was the arginase isoform expressed instead of Arg-1. We observed that infection of BMMΦ with the more virulent *T. cruzi* isolate (QRO) importantly diminished NOS2 expression and nitric oxide (NO) production in CAMΦ, allowing parasite survival, while infection with the less virulent isolate (CI2) did not diminish NOS2 activity and NO production in CAMΦ to a great extent, which resulted in parasite killing. Regarding Arg-1, infection of BMMΦ with the QRO isolate significantly induced Arg-1 expression and activity in AAMΦ, which resulted in a higher parasite load than the one in the unstimulated BMMΦ. Even though infection with CI2 isolate did not increase Arg-1 expression and activity in AAMΦ, the parasite load was higher than the one in the unstimulated BMMΦ but at a lesser magnitude than that observed during infection with the QRO isolate. On the other hand, infection of cardiomyocytes with either QRO or CI2 isolates and further stimulation with TNF-α + IFN-γ inhibited NOS2 expression and NO production, leading to amelioration of infection. Surprisingly, infection of cardiomyocytes with either QRO or CI2 isolates and further stimulation with IL-4 strongly inhibited Arg-2 expression and function, which resulted in parasite loads similar to those observed in unstimulated cardiomyocytes. Our results suggest that *T. cruzi* isolates that exhibit variable virulence or pathogenicity degrees differentially regulate L-arginine metabolism through Arg-1/2 and NOS2 in macrophages and cardiomyocytes.

## 1. Introduction

Chagas disease (CD), or American trypanosomiasis, is a vector-born neglected disease caused by the protozoan parasite *Trypanosoma cruzi* and represents an important public health problem worldwide. Although CD is endemic in 21 countries in Latin America, from Mexico to the south of Argentina and Chile, migrations and climate change have spread the disease to other parts of the world [1]. The infection of mammalian hosts, including humans, with *T. cruzi* parasites initiates through a bite, fissures in the skin, or mucous membranes when vectors (triatomines) defecate and expel metacyclic trypomastigotes in their droppings during a blood meal [2,3]. Initially, parasites invade macrophages and connective tissue cells close to the site of entry and later disseminate via lymph and blood to other tissues where they can infect any nucleated cell but most commonly macrophages, muscle cells (cardiac, smooth, and skeletal), and nervous cells. The cycle is completed when another vector ingests infected blood [4]. CD can transit through an initial acute phase that evolves within 1 to 2 weeks after the inoculation, lasts for 4–8 weeks, and is characterized by high levels of blood parasitemia or can progress to a chronic phase that can culminate in chronic cardiomyopathy in 30–40% of infected individuals, with a high risk of death due to heart failure associated with severe reactive myocardial fibrosis, cardiomegaly, and microvascular and electromechanical dysfunction [5,6,7,8]. Interestingly, this cardiac deterioration seems to be more associated with the host immune response and not with the parasite [9,10]. Nevertheless, the complete picture of factors involved in the development of the different phases of CD has not been unveiled as they result from complex interactions between the genetically diverse infecting *T. cruzi* and the host immune response to the infection. Regarding the diversity of *T. cruzi*, six discrete typification units (DTU TcI-VI) have been described and accepted by international consensus [11]. Among these, TcI is the lineage that has been described to possess the most diversity and wide distribution, the smallest genome, and the least amount of aneuploidy; TcV and TcVI are hybrid lineages, and TcBat is an additional lineage mainly found in bats and closely related to TcI [12,13]. Moreover, the main host defense against *T. cruzi* occurs via a T helper 1 (Th1) response [14], characterized by the production of cytokines such as IL-12, TNF-α, and IFN-γ, as well as nitric oxide (NO) [15] that have potent anti-trypanosomal effects. Contrarily, a Th2 phenotype, characterized by the production of IL-4 and IL-10 and the induction of arginase that initiates the catalysis of polyamines starting from L-arginine and concomitant reduction in NO synthesis, favors the parasitism of target organs [16,17,18,19]. Additionally, increased levels of IL-21, IL-23, and IL-1β induce a Th17 phenotype characterized by high IL-17 production [20], and the absence of this cytokine, along with Th1 cytokines, increases the host’s susceptibility to infection [10,21]. Therefore, host proneness or resistance to infection and the pathological outcome of Chagas disease is greatly modulated by the balance among the Th1, Th2, and Th17 profiles [22].

As already mentioned, a turning point in the development of CD is the regulation of the catabolism of L-arginine via inducible nitric oxide synthase (iNOS or NOS2) and arginase. Thus, *Trypanosoma* parasites can persist or die in host cells depending on the presence of NOS2 or arginase, whose expression is differentially induced and regulated in these cells. The best-studied model of reciprocal activation of these enzymes is found in one of the main *T. cruzi* host cells, macrophages. In classically activated macrophages (M1 or inflammatory macrophages), Th1 cytokines (e.g., IFN-γ and TNF-α) induce the expression and function of NOS2, which oxidizes L-arginine into L-citrulline and NO [23,24,25,26,27]. The latter is a free radical that mediates one of the most potent mechanisms to eliminate intracellular *T. cruzi* parasites [23]. On the other hand, Th2 cytokines, such as IL-4, IL-10, and IL-13, induce alternative activation of macrophages (M2 or wound-healing macrophages) characterized by the expression and function of arginase 1 (Arg-1) [23,24,25,26,27]. Other important host cells of the parasite are cardiomyocytes; whether these activation routes exerted by Th1 or Th2 cytokines have not been fully deciphered. Intriguingly, of the two arginase isoforms expressed in mammals, the cytosolic Arg-1 is abundantly present in macrophages, while the mitochondrial arginase 2 (Arg-2) is more expressed in cardiomyocytes. Both isoforms participate in the same reaction, which is the hydrolysis of L-arginine to urea and L-ornithine. Then, L-ornithine, through the enzyme ornithine decarboxylase, gives rise to polyamines putrescine, spermine, and spermidine, which play a pivotal role in cell homeostasis and tissue repair [28,29]. In the case of *Trypanosoma*, polyamines, particularly putrescine, along with trypanothione, have been shown to favor parasite growth in host cells [30]. Interestingly, for the production of polyamines, *Trypanosoma* parasites depend on the host arginase since they cannot generate their own source of ornithine through the activity of a functional arginase [31,32,33]. Several in vivo data have confirmed the relevant role that arginase plays in the infection with *Trypanosoma* by favoring parasite growth and thus counteracting the host’s immune response. The infection with *T. brucei gambiense*, a causal agent of African trypanosomiasis, elevates arginase levels in serum of patients that diminish after treatment [34]. In experimental murine trypanosomiasis, caused by *T. brucei*, arginase activity is induced in host myeloid cells by parasite excretion/secretion factors, particularly TbKHC1, a kinesin heavy chain isoform, and has been suggested as a disease susceptibility marker [35]. The importance of arginase for the growth of *Trypanosoma* has also been studied with inhibition approaches that have demonstrated that the addition of an arginase inhibitor reduces parasite growth, which is restored with L-ornithine supplementation. This result has led to the usage of a structural analog of L-ornithine, difluoromethylornithine, on its own or in combination with nifurtimox as an effective drug against African trypanosomiasis [36]. Unfortunately, its use in remote areas is difficult because its administration requires large amounts of i.v. injected fluids. That is why finding easier ways to target polyamine synthesis as a drug against *Trypanosoma* is crucial. Alternatively, parasite loads could be reduced with arginase inhibitors.

In the present study, we tested, in an in vitro murine model, the hypothesis that *T. cruzi* isolates with variable virulence degrees differentially regulate L-arginine metabolism through arginase and NOS2, influencing the infection course and severity in the host. Furthermore, we examined the participation of macrophages and cardiomyocytes and their activation status in this process.

## 2. Materials and Methods

### 2.1. Mice

CD1 mice were acquired from the Animal Facility of Instituto de Fisiología Celular, UNAM, and housed at the Animal Facility of the Unidad de Investigación UNAM-INC, Facultad de Medicina, UNAM. Mice were maintained following the guidelines of the Official Mexican Standard of technical specifications for the production, care, and use of laboratory animals (NOM-062-ZOO-1999) [37]. All the procedures practiced on mice during the study were previously approved by the Ethical Committee of the Facultad de Medicina, UNAM (approval number 114/2019).

### 2.2. Parasites

Two *Trypanosoma cruzi* isolates, genetically typified as DTU TcI, with important differences in infectivity (evaluated in in vitro assays) and in virulence (evaluated during mouse infections), were used in this study: QRO isolate (ITRI/MX/1986/QRO) and CI2 isolate (TcOAX-IST-CI2-TphM). The QRO isolate (more infective and virulent) was obtained from the vector *Triatoma barberi* in the Mexican state of Querétaro and kindly donated by Dr. Paz María Salazar-Schettino (Departamento de Microbiología y Parasitología, Facultad de Medicina, UNAM), while the CI2 isolate (less infective and virulent) was obtained from the vector *Triatoma phyllosoma* in the Mexican state of Oaxaca and kindly donated by Dr. Rebeca Manning-Cela (Departamento de Biomedicina Molecular, CINVESTAV, IPN). Trypomastigotes from both isolates were cultured in Vero cells according to Miles as well as Andrews and Colli [38,39] and were collected from supernatants of infected cell cultures.

### 2.3. Murine Bone Marrow-Derived Macrophage (BMMΦ) Culture

Macrophages were differentiated from mouse bone marrow cells cultured in the presence of macrophage colony-stimulating factor (M-CSF), as previously described [40], with minor modifications. Briefly, eight- to ten-week-old female CD-1 mice were euthanized by cervical dislocation, and hind legs were excised. Muscle and connective tissues were removed from femurs and tibias, and bone marrow was flushed out from the bone cavity by injecting PBS using a 25-gauge needle attached to a 10 mL syringe. Bone marrow suspension was centrifuged at 300× *g* for 10 min at room temperature and resuspended in DMEM: F12 medium (Biowest, Riverside, MO, USA) supplemented with 2 mM L-glutamine, 100 IU/mL penicillin, 100 µg/mL streptomycin (Mediatech Inc., Corning Inc., Corning, NY, USA), and 10% heat-inactivated fetal bovine serum (FBS) (Biowest, Riverside, MO, USA). Marrow cells were seeded at a density of 4 × 10^6^ leukocytes per 100 mm in bacteriological petri dishes (BD Falcon, Corning, Corning, NY, USA) in 10 mL of supplemented DMEM: F12 medium containing 20% of L-929 murine fibroblasts cell line culture supernatant as a source of M-CSF. Following four days of differentiation, 10 mL of freshly supplemented medium containing M-CSF was added to each petri dish. On day seven, mature BMMΦ were detached from dishes by incubation with PBS containing 5 mM EDTA for 10 min. Detached BMMΦ were seeded in supplemented DMEM: F12 medium without M-CSF in 12-well cell culture plates at a density of 1 × 10^6^ cells/well for Western blot experiments or in 24-well cell culture plates at a density of 5 × 10^5^ cells/well for NOS2 and arginase activity determination and at a density of 3 × 10^5^ cells/well for parasite load counting and allowed to rest overnight before infection and stimulation.

### 2.4. Neonatal Mouse Cardiomyocytes

Isolation and culture of neonatal mouse cardiomyocytes were performed as previously described [41,42], with minor modifications. One- to three-day-old neonatal CD1 mice were euthanized by cervical dislocation, and hearts were aseptically removed and placed in cold Hanks’ balanced salt solution without Ca^2+^ and Mg^2+^ (HBSS) (BioWest, Riverside, MO, USA). After two washes with HBSS, mouse hearts were minced into small pieces and predigested overnight in a mixture of 0.0125% Trypsin/0.11 mM EDTA (Mediatech, Inc.; Corning Inc, Corning, NY, USA) in HBSS, at 4 °C, with gentle agitation. The next day, Trypsin/EDTA solution was removed, and tissue fragments were digested two to three times (at 37 °C, with gentle agitation) in a collagenase type II solution (Worthington Biochemical Corp., Lakewood, NJ, USA) prepared at 350 units/mL in Leibovitz’s L-15 medium (Biowest, Riverside, MO, USA). Resultant cell suspensions from digestions were filtered through a 100 μm cell strainer (BD Falcon; Corning, Corning, NY, USA) and centrifuged at 180× *g* for 5 min. Cells were resuspended in 10 mL of DMEM medium supplemented with 2 mM L-glutamine, 100 IU/mL penicillin, 100 µg/mL streptomycin, and 10% FBS (Mediatech, Inc.; Corning Inc, Corning, NY, USA), plated in a 10 mm cell culture dish (Corning, Corning, NY, USA) and incubated for 2 h in a cell culture incubator to allow cardiac fibroblast and endothelial cells attachment. Following incubation, unattached cardiomyocytes were recovered from the culture dish and centrifuged at 180× *g* for 5 min. Cardiomyocytes were resuspended in supplemented DMEM, counted and plated in GelTrex matrix (Gibco; Thermo Fisher Scientific, Inc., Waltham, MA, USA)-coated 12-well cell culture plates at a density of 3.8 × 10^5^ cells/well for Western blot experiments and in GelTrex matrix-coated 24-well cell culture plates at a density of 1.9 × 10^5^ cells/well for NOS2 and arginase activity determination as well as for parasite load counting. Medium was changed 24 h after cardiomyocytes were plated and thereafter every three days. Finally, cardiomyocytes were used after seven to eight days of culture.

### 2.5. BMMΦ and Neonatal Mouse Cardiomyocytes Infection and Stimulation

Infection of BMMΦ and neonatal mouse cardiomyocytes with *T. cruzi* parasites was achieved by a 24 h exposure of both types of cells to trypomastigotes from QRO or CI2 isolates at a rate of five parasites per host cell (5:1) for experiments performed to evaluate changes in NOS2 and arginase expression and activity or two parasites per host cell (2:1) for experiments performed to determine parasite loads. Following the 24 h exposure to parasites, cells were washed three times with PBS and then stimulated for 48 h with either 20 U/mL of recombinant murine IL-4 (BD Pharmingen; BD Biosciences, Franklin Lakes, NJ, USA) or with a combination of 500 U/mL of recombinant murine TNF-α (PeproTech; Thermo Fisher Scientific, Inc., Waltham, MA, USA) and 100 U/mL of recombinant murine IFN-γ (BD Pharmingen; BD Biosciences, Franklin Lakes, NJ, USA). Uninfected and stimulated BMMΦ and neonatal mouse cardiomyocytes with IL-4 or TNF-α + IFN-γ were used as positive controls of the expression and activity of arginases and NOS2, respectively.

### 2.6. Western Blot

Infected and stimulated BMMΦ or neonatal mouse cardiomyocytes were washed twice with cold PBS and lysed in cold RIPA buffer (25 mM Tris-HCl pH 7.6, 150 mM NaCl, 1% NP-40, 1% sodium deoxycholate, and 0.1% SDS), containing 1× EDTA-free protease inhibitor cocktail (Roche Diagnostics Gmbh, Mannheim, Germany). Obtained lysates were clarified by centrifugation at 12,000× *g* for 15 min at 4 °C and protein concentration was quantified using the bicinchoninic acid (BCA) assay (Pierce; Thermo Fisher Scientific, Inc., Waltham, MA, USA). Equal amounts of protein (15 μg) were separated through SDS-PAGE (sodium dodecyl sulfate-polyacrylamide gel electrophoresis) using 10% acrylamide gels and transferred (at 100 V, for 60 min) onto Immobilon-P membranes (Millipore; Merck KGaA, Darmstadt, Germany) using a wet Mini Trans-Blot Cell Apparatus (Bio-Rad Laboratories, Hercules, CA, USA). Membranes were blocked for 30 min at room temperature with 5% nonfat dried milk (Bio-Rad Laboratories, Hercules, CA, USA) diluted in TBST (20 mM Tris-HCl pH 7.5, 150 mM NaCl, and 0.1% Tween-20). After blocking, membranes were incubated overnight at 4 °C with 1:10,000 monoclonal mouse anti-arginase-1 (Santa Cruz Biotechnology, Inc., Dallas, TX, USA), 1:10,000 polyclonal mouse anti-arginase-2 (GeneTex, Inc., Irvine, CA, USA), 1:5000 monoclonal rabbit anti-NOS2 (Cell Signaling Technology, Inc., Danvers, MA, USA), and 1:10,000 monoclonal mouse anti-β-tubulin (Invitrogen; Thermo Fisher Scientific, Inc., Waltham, MA, USA) primary antibodies diluted in TBST containing 5% nonfat dried milk. Following incubation with primary antibodies, membranes were washed three times with TBST (for 5 min each, with agitation) and then incubated for 60 min at room temperature with either 1:10,000 HRP-conjugated horse anti-mouse IgG or 1:10,000 HRP-conjugated goat anti-rabbit IgG secondary antibodies (Cell Signaling Technology, Inc., Danvers, MA, USA) diluted in TBS containing 5% nonfat dried milk. Following incubation with secondary antibodies, membranes were washed three times with TBST (for 5 min two times and one time for 10 min, with agitation), incubated with chemiluminescent substrate for HRP (Millipore; Merck KGaA, Darmstadt, Germany), and exposed to X-ray films (Santa Cruz Biotechnology, Inc., Dallas, TX, USA.). ImageJ software (Version 1.54j, Image Analysis Processing in Java rings; NIH, Bethesda, MD, USA) was used to perform densitometric analyses of bands.

### 2.7. Nitric Oxide Assay

Measurement of the concentration of nitrite (a stable product of nitric oxide metabolism) in cell culture supernatants using the Griess reaction was used to evaluate the activity of inducible nitric oxide synthase (NOS2). For this purpose, 100 μL of cell-free supernatants of infected and stimulated BMMΦ or neonatal mouse cardiomyocytes were mixed in 96-well flat-bottom plates with 100 μL of Griess reagent, which is made of equal volumes of 0.1% N-(1-naphtyl) ethylendiamine (NED’s) and 1% sulphanilamide in 5% phosphoric acid (all from Sigma-Aldrich; Merck KGaA, Darmstadt, Germany). After incubation for 10 min at room temperature, plates were read at 550 nm and nitrite concentration was determined using a standard curve performed with sodium nitrite.

### 2.8. Determination of Arginase Enzymatic Activity

Arginase enzymatic activity was determined in the infected and stimulated BMMΦ or neonatal mouse cardiomyocytes by measuring the amount of urea generated from the hydrolysis of L-arginine as previously described [43,44], with minor modifications. Cells were lysed in 300 μL of a buffer containing 25 mM Tris-HCl (pH 7.4), 0.1% Triton X-100. The lysates obtained were assayed for protein concentration using the bicinchoninic acid (BCA) assay. Next, a volume of 5–25 µL of cell lysates was diluted to a final volume of 50 µL with 25 mM Tris-HCl (pH 7.4), 0.1% Triton X-100 buffer supplemented with 10 mM MnCl_2_. Samples were then heated at 56 °C for 10 min to activate arginase enzyme, and 50 µL of L-arginine (0.5 M, pH 9.7) was added to each sample. L-arginine hydrolysis was conducted at 37 °C for 30–60 min. Hydrolysis was stopped by adding 400 µL of a mixture of H_2_SO_4_, H_3_PO_4_, H_2_O (1:3:7 *v*/*v*). The concentration of produced urea was measured at 550 nm, according to Archibald colorimetric reaction, after adding 20 µL of 7% α-isonitrosopropiophenone (dissolved in absolute ethanol) to each sample and heating at 100 °C for 30 min. Arginase activity was reported as units per mg of protein, and a unit of enzyme activity corresponds to the amount of enzyme that catalyzes the formation of 1 µmol of urea per minute.

### 2.9. Parasite Burden Assessment

Based on the knowledge that lysis of macrophages using low concentrations of sodium dodecyl sulfate (SDS) allows for other intracellular trypanosomatid parasites to release without affecting its viability [45,46,47], this method was used to assess *T. cruzi* parasite loads in infected macrophages and cardiomyocytes. Thus, BMMΦ or neonatal mouse cardiomyocytes were seeded in 24-well plates, infected, and stimulated as described above. Following, cells were washed three times with PBS and then incubated at 37 °C in 200 μL per well of a 0.01% SDS solution in PBS for 10–15 min to allow host cell lysis and intracellular *T. cruzi* amastigote release. Afterward, 800 μL of DMEM medium supplemented with 10% FBS was added to each well to neutralize SDS. Finally, resultant suspensions of parasites in the wells were resuspended by repeated pipetting, and the number of parasites per well was counted in a Neubauer chamber.

### 2.10. Data Analysis

Data are reported as the mean ± SEM (standard error of the mean) and were analyzed using GraphPad Prism 9.0.2 software (GraphPad Software, Inc., La Jolla, CA, USA). Statistical differences between groups were evaluated using one-way or two-way analyses of variance (ANOVA) followed by Tukey’s multiple comparisons post hoc test. Differences between groups were considered significant when *p*-value was ≤0.05.

## 3. Results

### 3.1. Characterization of BMMΦ and Neonatal Mouse Cardiomyocytes

**Figure 1:**
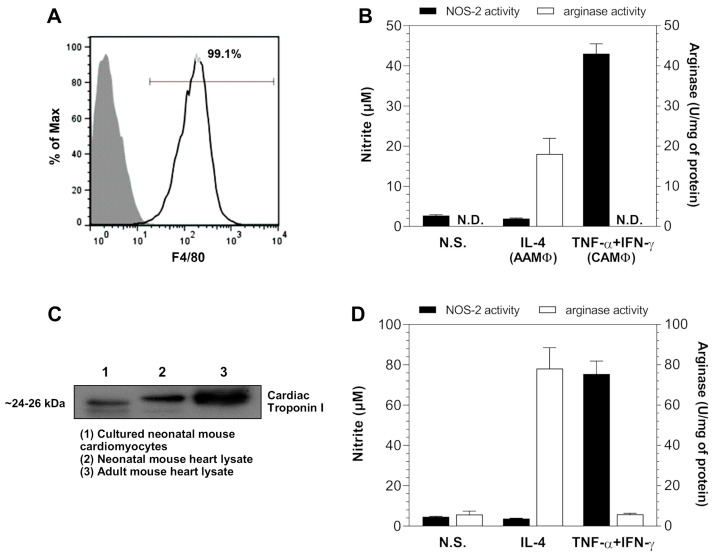
Bone marrow-derived macrophages (BMMΦ) and neonatal mouse cardiomyocytes characterization. BMMΦ obtained from CD1 mice were assayed by flow cytometry for their expression of the murine macrophage marker F4/80 after seven days of differentiation (**A**). BMMΦ were stimulated with TNF-α + IFN-γ to induce their classical activation (CAMΦ, classically activated macrophages) or with IL-4 to induce their alternative activation (AAMΦ, alternatively activated macrophages). At 48 h after stimulation, nitrite concentration (expressed in μM) in cell culture supernatants and arginase activity (expressed as U/mg of protein) in cell extracts were assessed (**B**). Neonatal mouse cardiomyocytes isolated from CD1 mice were analyzed by flow cytometry for their expression of troponin I after seven or eight days of culture; heart lysates from neonatal and adult CD1 mice were used as controls (**C**). Neonatal mouse cardiomyocytes were stimulated for 48 h with TNF-α + IFN-γ or IL-4, and nitrite concentration and arginase activity were assessed (**D**). N.S., non-stimulated; N.D., not detected. Data are expressed as mean ± SEM of 3 independent experiments.

Bone marrow macrophages (BMMΦ) were differentiated from bone marrow cells from CD1 mice cultured in the presence of macrophage colony-stimulating factor (M-CSF). On day seven of culture, differentiated BMMΦ expressed the murine macrophage marker F4/80 (Figure 1A). BMMΦ were stimulated for 48 h with TNF-α + IFN-γ or IL-4 to polarize them into classically activated macrophages (CAMΦ) and alternatively activated macrophages (AAMΦ), respectively. The correct polarization of BMMΦ into CAMΦ was demonstrated by their production of nitric oxide, quantified indirectly through the measurement of nitrite (a stable breakdown product of NO) in the cell culture supernatants (Figure 1B). The correct polarization of BMMΦ into AAMΦ was demonstrated by their induced arginase activity, measured as the amount of urea produced from the hydrolysis of L-arginine in cell lysates (Figure 1B). Neonatal mouse cardiomyocytes were isolated from the hearts of newborn CD1 mice and maintained in culture for seven to eight days, when they were shown to express troponin I (Figure 1C). Neonatal mouse cardiomyocytes were also stimulated for 48 h with TNF-α + IFN-γ or IL-4, and like CAMΦ and AAMΦ, they produced NO and increased their arginase activity, respectively (Figure 1D).

### 3.2. NOS2 and Arg-1 Expression and Activity Are Differentially Regulated in CAMΦ and AAMΦ, Respectively, Infected with T. cruzi Isolates with Different Virulence Degrees

**Figure 2:**
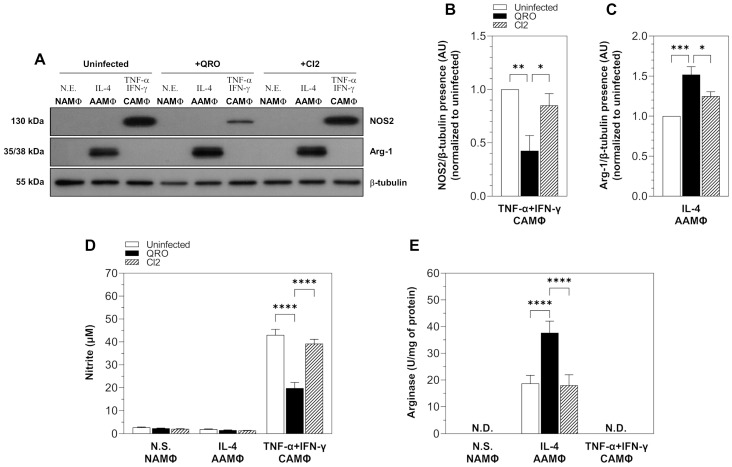
NOS2 and Arg-1 expression and activity are differentially regulated in CAMΦ and AAMΦ, respectively, infected with *T. cruzi* isolates with different virulence degrees. BMMΦ were infected with *T. cruzi* isolates with different virulence degrees (QRO, more virulent; CI2, less virulent) for 24 h and then polarized into CAMΦ and AAMΦ by stimulation for 48 h with TNF-α + IFN-γ or IL-4, respectively. Uninfected macrophages and non-stimulated, N.S., macrophages (non-activated macrophages, NAMΦ) were used as controls. Cells were lysed, and NOS2 and Arg-1 expression were assessed by Western blot using β-tubulin expression as loading control (**A**). Densitometric values (expressed in arbitrary units, A.U) for bands corresponding to NOS2 (**B**) and Arg-1 (**C**) expression were calculated as the ratio of band intensities for NOS2 or Arg-1 and β-tubulin detected in the cell lysates. (**D**) NOS2 activity was determined by the quantification of nitrite present in the culture supernatants of BMMΦ uninfected and infected with *T. cruzi* isolates and polarized into CAMΦ and AAMΦ. (**E**) Arg-1 activity was determined by enzymatic assay in cell lysates of BMMΦ uninfected and infected with *T. cruzi* isolates and polarized into CAMΦ and AAMΦ. N.D., not detected. Data are expressed as mean ± SEM of 3 independent experiments (* *p* < 0.05; ** *p* ≤ 0.01; *** *p* < 0.0001; **** *p* < 0.00001).

It has been demonstrated that macrophages stimulated with cruzipain exhibit enhanced arginase activity [18,19], which favors parasite multiplication and spreading. On the other hand, NOS2 presence has been correlated with parasite clearance and disease resolution in *T. cruzi* infections [23,24,25,26,27]. Thus, we analyzed if infection with two *T. cruzi* isolates with different degrees of virulence resulted in a differential expression and activity of NOS2 and Arg-1 in BMMΦ of CD1 mice. For this purpose, BMMΦ were infected with both *T. cruzi* isolates for 24 h and then polarized into CAMΦ or AAMΦ by stimulation for 48 h with TNF-α + IFN-γ and IL-4, respectively. Afterward, cells were lysed, and NOS2 and Arg-1 expression was detected by Western blot (Figure 2A). The assays demonstrated the presence of a 130 kDa band corresponding to NOS2 expression in CAMΦ, which diminished when they were infected with the more virulent *T. cruzi* isolate (QRO) (Figure 2A,B). In contrast, CAMΦ infected with the less virulent *T. cruzi* isolate (CI2) exhibited similar NOS2 expression levels to those observed in uninfected CAMΦ (Figure 2A,B). On the other hand, Western blot assays showed the presence of two bands with molecular weights of 35 and 38 kDa, corresponding to Arg-1 isoforms expression in AAMΦ, which increased when they were infected with the more virulent *T. cruzi* isolate (QRO). Contrarily, the infection of AAMΦ with the less virulent *T. cruzi* isolate (CI2) resulted in a similar Arg-1 expression compared to the one observed in uninfected AAMΦ (Figure 2A,C). Once we determined that the *T. cruzi* isolates differentially modulated NOS2 expression in CAMΦ and Arg-1 expression in AAMΦ, we evaluated if this modulation paralleled enzyme activity. For this aim, NOS2 and Arg-1 activity were determined in the culture supernatants or cell extracts, respectively, of BMMΦ infected with both *T. cruzi* isolates and polarized into CAMΦ and AAMΦ (Figure 2D,E). Obtained data revealed that CAMΦ produced a significant amount of nitrite (more than 45 μM) that drastically diminished (14 μM) when they were infected with the QRO isolate. Conversely, infection of CAMΦ with the CI2 isolate resulted in the production of nitrite levels similar to those produced by uninfected CAMΦ (Figure 2D). Regarding Arg-1 activity, our data demonstrated the induction of arginase activity in AAMΦ increased by almost twice when they were infected with the QRO isolate, while infection with the CI2 isolate resulted in arginase activity similar to that observed in uninfected AAMΦ (Figure 2E).

### 3.3. NOS2 and Arg-2 Expression and Activity Are Down-Regulated in Neonatal Mouse Cardiomyocytes Infected with T. cruzi Isolates with Different Virulence Degrees

**Figure 3:**
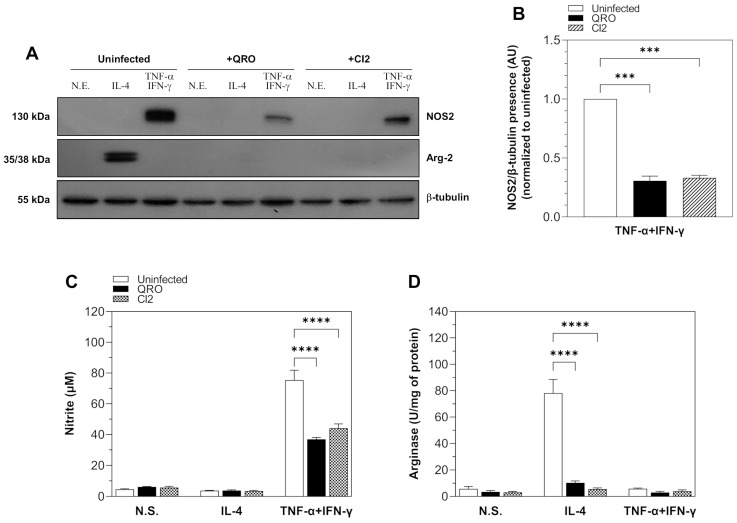
NOS2 and Arg-2 expression and activity are down-regulated in neonatal mouse cardiomyocytes infected with *T. cruzi* isolates with different virulence degrees. Neonatal mouse cardiomyocytes were infected with *T. cruzi* isolates with different virulence degrees (QRO, more virulent; CI2, less virulent) for 24 h and then were stimulated for 48 h with TNF-α + IFN-γ or IL-4, respectively. Uninfected and non-stimulated, N.S., cardiomyocytes were used as controls. Cells were lysed, and NOS2 and Arg-2 expression was assessed by Western blot using β-tubulin expression as loading control (**A**). Densitometric values (expressed in arbitrary units, A.U) for bands corresponding to NOS2 (**B**) expression were calculated as the ratio of band intensities for NOS2 and b-tubulin detected in the cell lysates. (**C**) NOS2 activity was determined by the quantification of nitrite present in the culture supernatants of neonatal mouse cardiomyocytes uninfected and infected with *T. cruzi* isolates and stimulated with TNF-α + IFN-γ or IL-4. (**D**) Arg-1 activity was determined by enzymatic assay in cell lysates of neonatal mouse cardiomyocytes uninfected and infected with *T. cruzi* isolates and stimulated with TNF-α + IFN-γ or IL-4. Data are expressed as mean ± SEM of 3 independent experiments (*** *p* < 0.0001; **** *p* < 0.00001).

Contrary to macrophages, where the reciprocal activation of NOS2 and Arg-1 has been extensively studied, this metabolic pathway has been poorly investigated in cardiac cells. We aimed to analyze the effect of both *T. cruzi* isolates in the expression and activity of NOS2 and Arg-2 in cardiomyocytes obtained from neonatal CD1 mice and stimulated with TNF-α + IFN-γ or IL-4. Interestingly, our results show that neonatal mouse cardiomyocytes have the ability to abundantly express NOS2 when stimulated with TNF-α + IFN-γ and conversely express Arg-2 when stimulated with IL-4 (Figure 3A). This is different from what occurs in macrophages, in which the arginase isoform expressed as a result of stimulation with IL-4 is Arg-1. We found that infection of neonatal mouse cardiomyocytes with both *T. cruzi* isolates with different virulence degrees, followed by stimulation with TNF-α + IFN-γ, led to a significant decrease in NOS2 expression, which was similar in magnitude (Figure 3A,B). Regarding Arg-2 expression, even though it was induced in neonatal mouse cardiomyocytes stimulated with IL-4, interestingly, the infection with both *T. cruzi* isolates diminished the expression of the enzyme to the extent that it was not possible to detect it by Western blot (Figure 3A). Once we determined that infection with the *T. cruzi* isolates inhibited NOS2 and Arg-2 expression in neonatal mouse cardiomyocytes, we evaluated if this inhibition at the protein expression level paralleled enzyme activity. For this aim, NOS2 and Arg-2 activity was determined in the culture supernatants or cell extracts, respectively, of neonatal mouse cardiomyocytes infected with both *T. cruzi* isolates and stimulated with TNF-α + IFN-γ or IL-4. We found that neonatal mouse cardiomyocytes stimulated with TNF-α + IFN-γ produced even more nitrite than BMMΦ (more than 70 μM), and this production noticeably diminished with the infection with both *T. cruzi* isolates (Figure 3C). We did not find statistically significant differences between infection with both isolates (Figure 3C). Furthermore, neonatal mouse cardiomyocytes stimulated with IL-4 showed an increased arginase activity (more than 75 U/mg of protein), which diminished significantly and in a similar magnitude during infection with either of the *T. cruzi* isolates (Figure 3D).

### 3.4. AAMΦ and CAMΦ Differentially Host or Eliminate, Respectively, T. cruzi Parasites of Isolates with Different Virulence Degrees, While Neonatal Mouse Cardiomyocytes Stimulated with TNF-α + IFN-γ Equally Eliminates T. cruzi Parasites of Isolates with Different Virulence Degrees 

**Figure 4:**
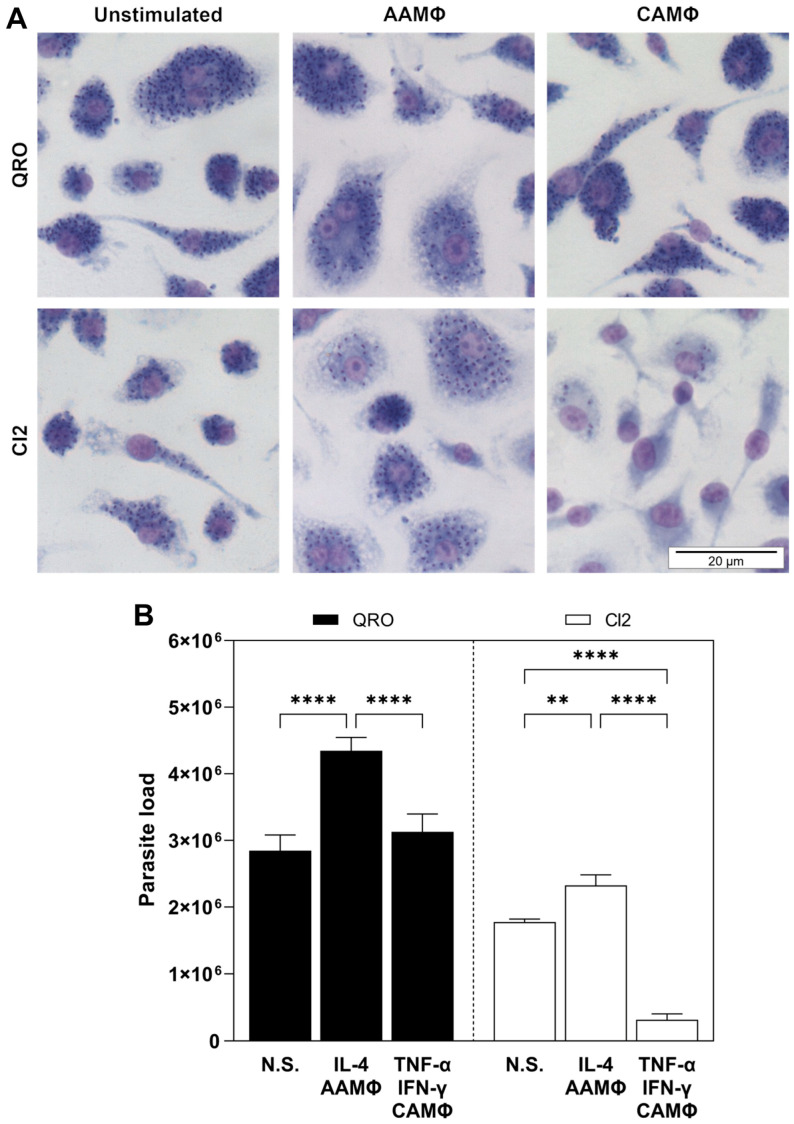
AAMΦ and CAMΦ differentially host or eliminate, respectively, *T. cruzi* parasites of isolates with different virulence degrees. (**A**) Micrographs of Giemsa-stained BMMΦ infected with *T. cruzi* parasites of isolates with different virulence degrees (QRO, more virulent; CI2, less virulent) and non-stimulated (N.S.) and stimulated with IL-4- or TNF-α + IFN-γ to polarize them into alternatively activated macrophages (AAMΦ) and classically activated macrophages (CAMΦ), respectively. Bars = 20 μm. Micrographs are representative of 3 independent experiments. (**B**) Infected and stimulated BMMΦ were lysed with SDS, and released parasites were counted in a Neubauer chamber to determine parasite load. Data are expressed as mean ± SEM of triplicate samples, and similar results were observed from 3 independent experiments (** *p* ≤ 0.01; **** *p* < 0.00001).

**Figure 5:**
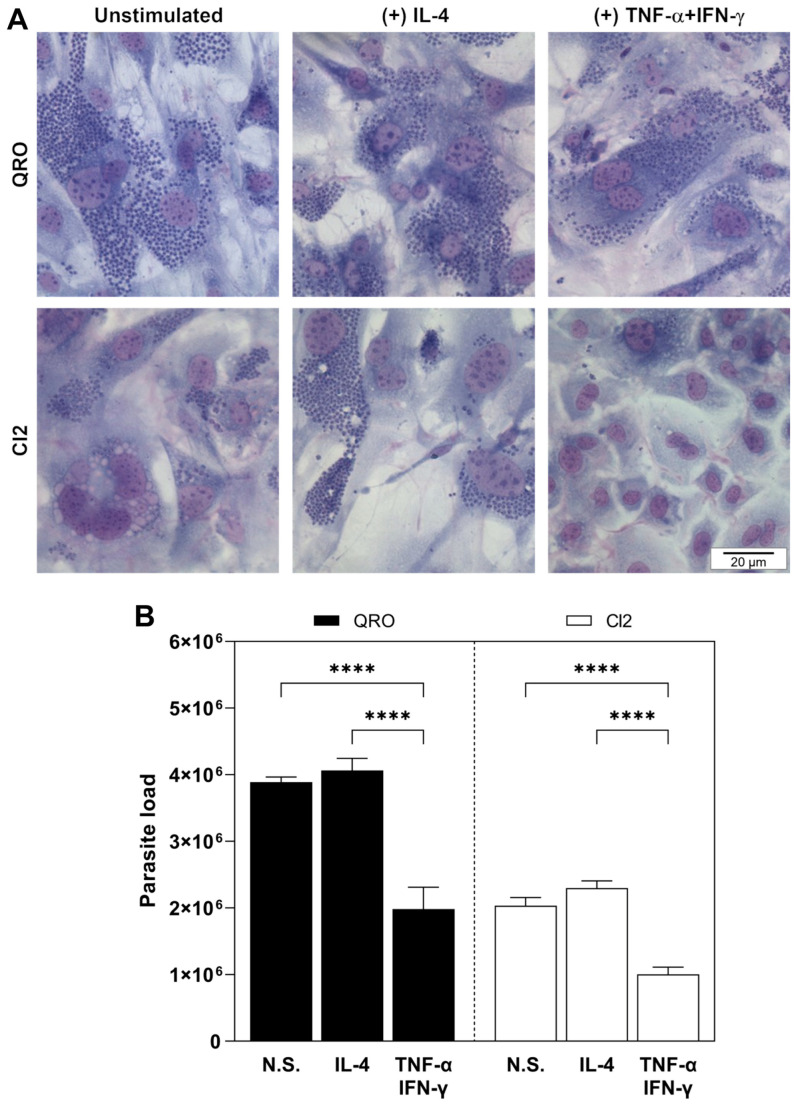
Neonatal mouse cardiomyocytes stimulated with TNF-α + IFN-γ equally eliminate *T. cruzi* parasites of isolates with different virulence degrees. (**A**) Micrographs of Giemsa-stained neonatal mouse cardiomyocytes infected with *T. cruzi* parasites of isolates with different virulence degrees (QRO, more virulent; CI2, less virulent) and non-stimulated (N.S.) and stimulated with IL-4- or TNF-α + IFN-γ. Bar = 20 μm. Micrographs are representative of 3 independent experiments. (**B**) Infected and stimulated neonatal mouse cardiomyocytes were lysed with SDS, and released parasites were counted in a Neubauer chamber to determine parasite load. Data are expressed as mean ± SEM of triplicate samples, and similar results were observed from 3 independent experiments (**** *p* < 0.00001).

Once we determined that the *T. cruzi* isolates with different virulence degrees differentially modulated NOS2 and Arg-1 expression and activity in CAMΦ and AAMΦ and that down-regulated NOS2 and Arg-2 expression and activity in neonatal mouse cardiomyocytes, we aimed to demonstrate how these regulations affected parasite survival in these two host cells. To demonstrate the infection of BMMΦ and neonatal cardiomyocytes, these were adhered to glass coverslips and infected for 24 h with *T. cruzi* trypomastigotes of the QRO (more virulent) or CI2 (less virulent) isolates at a parasite/cell ratio of 2:1 and afterward were stimulated with TNF-α + IFN-γ or IL-4 for 48 h. Then, cells were fixed with methanol, stained with Giemsa, mounted on microscope slides, and observed in a compound optical microscope (Figure 4 4A and Figure 5A). Parasite loads in BMMΦ and neonatal mouse cardiomyocytes were assessed by counting in a Neubauer chamber the parasites released from cells after lysis with SDS. As shown in Figure 4B, the QRO isolate was more infective than the CI2 isolate in both BMMΦ and neonatal mouse cardiomyocytes, which was demonstrated by the detection of higher parasite loads in unstimulated cells (Figure 4B and Figure 5B). Interestingly, AAMΦ, as result of the stimulation of infected BMMΦ with IL-4, harbored higher numbers of parasites of both isolates than the unstimulated BMMΦ (Figure 4B). This behavior was observed to a major extent during infection with the QRO isolate (Figure 4B). Contrarily, CAMΦ, as result of the stimulation of infected BMMΦ with TNF-α + IFN-γ, diminished parasite load only when they were infected with the CI2 isolate, while CAMΦ infected with the QRO isolate were not capable of diminishing parasite load, exhibiting similar parasite numbers to those of unstimulated BMMΦ (Figure 4B). Regarding neonatal mouse cardiomyocytes, infection with either of the isolates and stimulation with IL-4 did not significantly increase parasite loads (Figure 5B). Moreover, stimulation with TNF-α + IFN-γ of neonatal mouse cardiomyocytes infected with any of the isolates effectively diminished parasite loads to a similar extent (Figure 5B).

## 4. Discussion

In the present study, we examined, in an in vitro model, the ability of two *T. cruzi* isolates, with variable virulence degrees, to differentially regulate the L-arginine metabolism through Arg-1, Arg-2, and NOS2. The rationale for performing the study in vitro was firstly to investigate the effect of the infection of the main *Trypanosoma cruzi* host cells, macrophages, and cardiomyocytes with isolates with different virulence degrees and secondly to determine the effect of polarizing both cell types with Th1 or Th2 cytokines and determine the fate of the parasite in the polarized cells. We used primary cultures, which undoubtedly resemble more in vivo conditions than cell lines. In our model, we obtained macrophages and cardiomyocytes from CD1 mice due to the high susceptibility of this mouse strain to *T. cruzi* infection infected with the isolates, polarized them to a M1/M2-type phenotype, and determined the expression and activity of NOS-2 and Arg-1/2. Although cardiomyocytes are one of the major target cells of Chagas disease, knowledge about myocardial tissue response to local parasite presence and consequent inflammation is scarce. On the other hand, the response of macrophages to *T. cruzi* has been documented to a greater extent, as well as the wide spectrum of activation states that these cells present. Firstly, we characterized the cell populations and the effect that the stimulation with Th1 or Th2 cytokines exerted on them. We showed a very pure and uniform population of bone marrow-derived macrophages (BMMΦ) demonstrated by the expression of F4/80. The stimulation of these cells with TNF-α + IFN-γ achieved the activity of NOS2, while the stimulation with IL-4 showed Arg-1 activation. Moreover, cardiomyocytes obtained from neonatal CD1 mice were characterized by the presence of troponin. In a fascinating way, when neonatal mouse cardiomyocytes were stimulated with TNF-α + IFN-γ or IL-4, we found that these cytokines drove the phenotype of cardiomyocytes to an M1/M2-type phenotype as has extensively been documented in macrophages [24,25,26,27]. Some reports have documented that IL-4 also increases arginase activity in myocardial cells or smooth muscle cells [28,48]. In this study, we found that IL-4 strongly stimulates the L-arginine metabolic pathway mediated by arginase in neonatal mouse cardiomyocytes. Interestingly, the isoform of arginase present in BMMΦ stimulated with IL-4 was Arg-1, while the isoform of arginase present in neonatal mouse cardiomyocytes stimulated with IL-4 was Arg-2. Two arginase isoforms, encoded by different genes, are expressed in mammals: the cytosolic arginase-1 and the mitochondrial arginase-2. Both isoforms catalyze the same reaction but differ in their tissue distribution [49]. Arg-1 is the fifth enzyme of the urea cycle, is abundantly expressed in the liver, and is responsible for most of the total body arginase activity [29]. On the other hand, Arg-2 has a lower expression in several tissues [50,51], and its physiological role has not been well established as in the case of Arg-1. Once we characterized the cell populations and the phenotype obtained with the stimulation with TNF-α + IFN-γ or IL-4, we proceeded to analyze the effect of the infection of BMMΦ and neonatal mouse cardiomyocytes with two different *T. cruzi* isolates in the modulation of L-arginine metabolism. The rationale for evaluating this was that the fate of L-arginine in *T. cruzi* host cells has echo in the development of the disease, and probably *T. cruzi* isolates with variable virulence degrees differently modulate this metabolic pathway. The isolates that we tested were QRO and CI2. The QRO isolate was obtained from the vector *Triatoma barberi* in the Mexican state of Querétaro. The CI2 isolate was obtained from the vector *Triatoma phyllosoma* in the Mexican state of Oaxaca. The QRO isolate is considered a highly virulent one due to its elevated infectivity and pathogenicity assessed in a murine experimental infection model [52]. On the other hand, the CI2 isolate has proven to be a less virulent one during infection in mice and a less infective one in in vitro assays performed in different cell types [unpublished results]. We infected BMMΦ and neonatal mouse cardiomyocytes with these isolates, then stimulated them with TNF-α + IFN-γ or IL-4 and determined the expression of NOS2 and Arg and the activity of both enzymes. In the case of BMMΦ, the infection with *T. cruzi* and stimulation with TNF-α + IFN-γ diminished NO2 protein presence, and interestingly, the infection with the QRO isolate diminished NOS2 protein presence to a major extent as compared to the infection with the CI2 isolate. We expected to find a decrease in NOS2 due to the infection since *T. cruzi* needs to down-regulate this enzyme on its behalf to prevent the action of trypanocydal NO and its derivatives. It was remarkable to find out that the more virulent QRO isolate had a major effect on this down-regulation. The activity of NOS2, demonstrated by the Griess assay, showed a concordant result where *T. cruzi* infection diminished the production of nitrites after the stimulation of cells with TNF-α + IFN-γ and the QRO isolate was more effective in diminishing the production of nitrites compared to the CI2 isolate. In terms of the modulation of NOS2 by *T. cruzi* in neonatal mouse cardiomyocytes, cultures stimulated with TNF-α + IFN-γ resulted in a significant increase in NO levels, similar to previously shown results [53]. It has been postulated that the strong NOS induction elicited by this treatment leads cardiomyocytes to apoptosis [54]. When neonatal mouse cardiomyocytes were infected with *T. cruzi* isolates and stimulated with TNF-α + IFN-γ, there was an important decrease in the expression and activity of NOS2, and the effect of the infection with both isolates was similar. On the other hand, the polarization of BMMΦ to an M2 phenotype with IL-4 produced the expected effect of induction of Arg-1, which was upregulated when BMMΦ were infected with *T. cruzi* and stimulated with IL-4. The increase in Arg-1 protein presence was more evident with the QRO isolate when compared to the CI2. Actually, the CI2 isolate did not upregulate Arg-1 activity. The result with the QRO isolate was expected due to the importance of host arginase for the production of polyamines for the duplication of *T. cruzi*. In particular, putrescine has been clearly implicated in parasite growth [30], although *T. cruzi* is incapable of producing this polyamine because it lacks both ornithine decarboxylase (ODC) and arginase. Thus, it needs to capture exogenous putrescine for the proliferation of amastigote forms within the cells [55,56]. Regarding the role of *T. cruzi* infection in the modulation of Arg-2 in neonatal mouse cardiomyocytes, we obtained an unexpected result. The infection of cardiomyocytes with *T. cruzi* isolates, and stimulation with IL-4 did not produce the anticipated effect. Rather than induction in Arg-2 protein presence and activity, we observed an important down-regulation. We were not able to detect Arg-2 protein presence in infected neonatal mouse cardiomyocytes and stimulated with IL-4 and the activity drastically diminished in infected cells. This result differs from what has been described by Aoki and cols. Moreover, [57] that showed that Arg-2 is upregulated in cardiomyocytes primary cultures after cruzipain treatment. They found a basal level of arginase activity in cardiomyocyte cultures corresponding to both arginase isoforms, but only Arg-2 expression was increased by cruzipain [57]. Other authors have also shown that arginase expression and activity are strongly induced in the heart tissue of mice infected with *T. cruzi*. They found that the cellular source of Arg-2 was cardiomyocytes and infiltrating macrophages of Arg-1 [58]. The differences between our result and previous observations may be due in part to the fact that we infected a purified cell population instead of analyzing the organ and with whole parasites and not with a molecule. The down-regulation of Arg-2 observed in infected neonatal mouse cardiomyocytes with two different *T. cruzi* isolates and stimulation with IL-4 suggests that in cardiomyocytes, the parasite is not taking advantage of the arginase-induced turnover of L-arginine to produce polyamines, which are necessary for its growth and replication. The down-regulation of Arg-2 activity in neonatal mouse cardiomyocytes that we observed may be involved in the damage caused in the heart during chronic chagasic cardiomyopathy. It is currently believed that Arg-2 exerts a protective effect in organ damage, as has been shown in renal ischemia-reperfusion injury (IRI) [59]. In rats exposed to IRI, it has been shown that Arg-2 protects from myocardium lesions and cardiomyocyte apoptosis [60].

After determining the effect of the infection of BMMΦ and neonatal mouse cardiomyocytes with two different *T. cruzi* isolates in the metabolism of L-arginine through NOS2 and arginases, we proceeded to analyze the effect of the enzymes in the survival of parasites inside the cells. We demonstrated, qualitatively, the infection of the cells and the effect of the enzymes in micrographs of infected and stimulated cells stained with Giemsa. As observed in the micrographs, the number of parasites inside the cells is so large that it is impossible to quantify them. To achieve quantification of the parasites, we released them from the cells with a light treatment with SDS and counted them in a Neubauer chamber. Noticeably, the QRO isolate was more infective in both BMMΦ and neonatal mouse cardiomyocytes than the CI2 isolate, as shown by a larger parasite load obtained both in BMMΦ and neonatal mouse cardiomyocytes. Interestingly, the stimulation with TNF-α + IFN-γ of CI2-infected BMMΦ was highly effective in the elimination of the parasites and not effective in the case of infection with the QRO isolate. The stimulation of infected BMMΦ with IL-4 increased the levels of Arg-1 to a major extent with the QRO isolate, which correlated with an increase in the parasite load. Contrarily, the stimulation of infected neonatal mouse cardiomyocytes with IL-4 did not significantly increase the parasite loads.

In conclusion, our results highlight the important role of L-arginine metabolism through NOS2 and Arg-1/2 during infection with *T. cruzi*. Specifically, the evidence shown here tempt us to suggest a differential regulation of this novel pathway during the infection of both BMMΦ and neonatal mouse cardiomyocytes with *T. cruzi* isolates with variable virulence degrees. Our findings provide new insights into the complex regulation of the still rather unknown family of enzymes involved in the L-arginine metabolic pathways and should help to clarify the functional importance of arginase within cardiovascular physiology. Undoubtedly, further studies involving experimentation with other isolates obtained from patients, gene expression profile identification during experimental and human infection, and evaluation of new parameters will be needed to strengthen our findings.

## Data Availability

The original contributions presented in the study are included in the article. Further inquiries can be directed to the corresponding author.
